# A phase I study of S-1 and cisplatin with concurrent hypofractionated carbon-ion radiotherapy for patients with stage III non-small cell lung cancer

**DOI:** 10.3389/fonc.2025.1573462

**Published:** 2025-08-05

**Authors:** Yosuke Miura, Nobuteru Kubo, Noriaki Sunaga, Naoko Okano, Hiroaki Tsurumaki, Hidemasa Kawamura, Reiko Sakurai, Toshitaka Maeno, Takeshi Hisada, Tatsuya Ohno

**Affiliations:** ^1^ Department of Respiratory Medicine, Gunma University Graduate School of Medicine, Maebashi, Gunma, Japan; ^2^ Gunma University Heavy Ion Medical Center, Maebashi, Gunma, Japan; ^3^ Department of Medical Oncology, Gunma University Graduate School of Medicine, Maebashi, Gunma, Japan; ^4^ Gunma University Graduate School of Health Sciences, Maebashi, Gunma, Japan

**Keywords:** carbon-ion radiotherapy, concurrent chemoradiotherapy, non-small cell lung cancer, platinum-based chemotherapy, clinical trial

## Abstract

**Objectives:**

We conducted a phase I study to evaluate the recommended dose of S-1 in combination with cisplatin (SP) and concurrent carbon ion radiotherapy (CIRT) in patients with stage III locally advanced non-small cell lung cancer (LA-NSCLC).

**Materials and methods:**

S-1 was administered orally twice daily after a meal for 14 consecutive days, and cisplatin was administered on days 1 and 8. The dose of each drug in this study was planned as follows: level 0, S-1–30 mg/m^2^ twice daily and cisplatin 40 mg/m^2^; level 1, S-1–40 mg/m^2^ twice daily and cisplatin 40 mg/m^2^. CIRT was conducted at a total dose of 64 Gy (relative biological effectiveness) in 16 fractions.

**Results:**

Six patients were enrolled in this study. At level 1, one patient experienced grade 3 elevated alanine aminotransferase and aspartate aminotransferase levels, which is regarded as a dose-limiting toxicity. This event improved immediately. Five patients developed grade 2 esophagitis. In three of the five patients, symptoms such as pain and dysphagia due to esophagitis recurred several months after resolution of the acute esophagitis that occurred during irradiation. None of the patients experienced adverse events of ≥grade 3. Thus, level 1 was determined to be the recommended dose.

**Conclusion:**

Chemotherapy with SP and concurrent CIRT is feasible and well-tolerated in patients with Stage III LA-NSCLC.

## 1 Introduction

Lung cancer is a common cause of cancer-related mortalities worldwide. The most common histological type is non-small cell lung cancer (NSCLC), which accounts for approximately 85% of all lung cancer cases. Approximately 25% of all NSCLC cases are diagnosed as unresectable locally advanced NSCLC (LA-NSCLC) (1). Currently, the standard treatment is platinum-based chemotherapy with concurrent thoracic radiotherapy followed by consolidation therapy with durvalumab. Consolidative treatment with durvalumab after concurrent chemoradiation therapy (cCRT) has significantly improved clinical outcomes compared to chemoradiation therapy (CRT) alone (2). However, 5-year progression-free survival (PFS) and overall survival (OS) rates of 33.1% and 42.9%, respectively, in patients with stage III LA-NSCLC treated with cCRT and consolidation durvalumab therapy remain unsatisfactory (3).

Compared to X-ray radiotherapy, carbon ion radiotherapy (CIRT) provides a higher conformal dose distribution to the tumor, sparing normal tissues (4). It has higher linear energy transfer, resulting in greater relative biological effectiveness (RBE) (5). CIRT without chemotherapy has been reported for patients with LA-NSCLC. Although 2-year local control rates are excellent (73.9–100%), the median survival time is 24–27.6 months due to distant metastasis (6–8). Several preclinical studies have suggested that anticancer agents can sensitize human NSCLC cells to carbon ion irradiation (9, 10). However, the effect of combined CIRT and chemotherapy in patients with LA-NSCLC remains unclear.

S-1 plus cisplatin (SP) is a great candidate for chemotherapy regimens combined with concurrent CIRT. Several phase II studies have demonstrated that the 2-year OS rate in patients undergoing cCRT with SP was 52.9–75.6% (11–14), equivalent to that of cCRT with standard chemotherapy regimens. Additionally, cCRT with SP is reportedly more feasible than cCRT with conventional chemotherapy (12).

Therefore, we conducted a phase I study to evaluate the recommended dose of SP and concurrent CIRT in patients with stage III LA-NSCLC.

## 2 Materials and methods

### 2.1 Patient eligibility

The eligibility criteria for this study were as follows: histologically or cytologically proven unresectable stage III NSCLC (Union for International Cancer Control 8th edition) (15); a performance status (PS) of 0 or 1 on the Eastern Cooperative Oncology Group (ECOG) scale; ages between 20 and 74 years; life expectancy of ≥3 months; adequate bone marrow activity (neutrophil count ≥1500 mm^-3^, hemoglobin ≥9.0 g/dL, and platelet count ≥100,000 mm^-3^); aspartate transaminase [AST] and alanine transaminase [ALT] ≤2.5 times the upper limit of the normal range; total serum bilirubin ≤1.5 times the upper limit of the normal range; estimated glomerular filtration rate ≥60 ml/min/m^2^; and oxygen saturation ≥90%. Patients were excluded if they had any of the following: malignant pericardial or pleural effusions; interstitial lung disease; active double cancer; concomitant serious illness such as uncontrolled angina pectoris; myocardial infarction in the previous 3 months; tumor invasion to the heart, large vessels, trachea, or esophagus; metastases to contralateral hilar lymph nodes; infection or other diseases contraindicating chemotherapy or radiotherapy; pregnancy; or breastfeeding. This study was approved by the local institutional ethics committee, and written informed consent was obtained from all patients. This study was registered with the Japan Registry of Clinical Trials (jRCTs031190126).

### 2.2 Study design

#### 2.2.1 Chemotherapy

**Figure 1:**
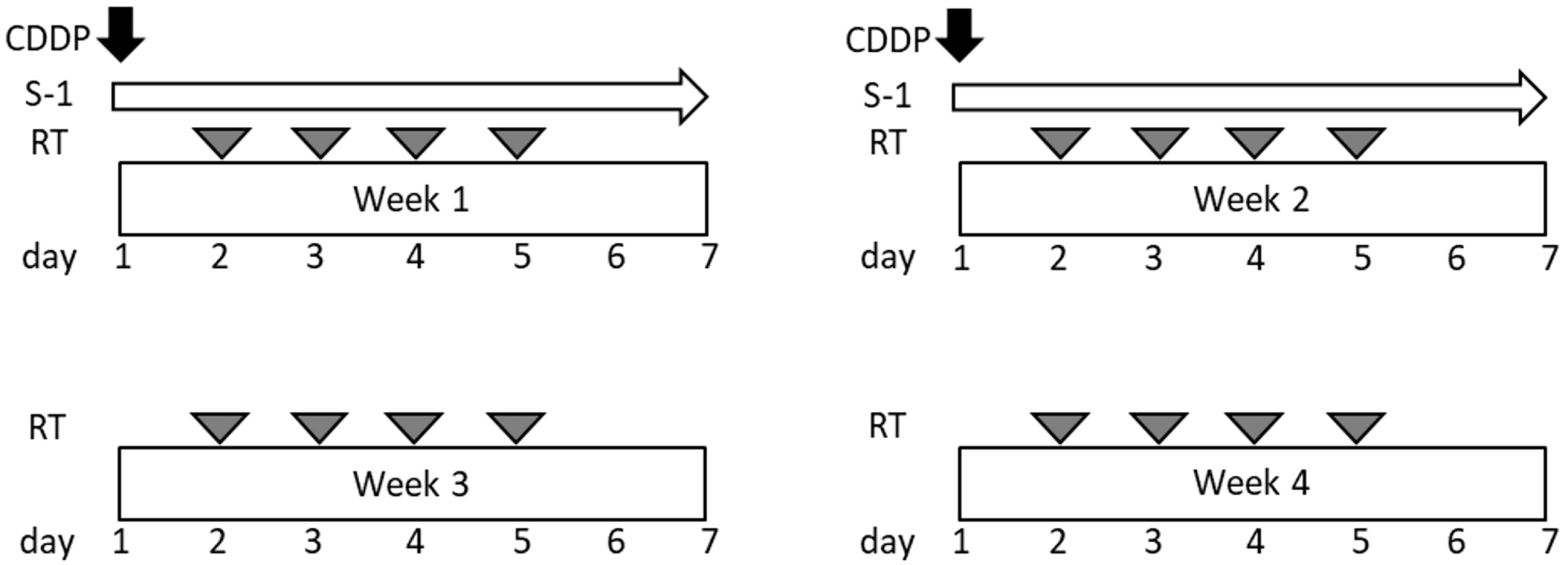
Scheme of the protocol treatment.

This was an open-label, single-center, single-arm, phase I study. The chemotherapy regimen consisted of one cycle of S-1 and cisplatin (**Figure 1**). S-1 was orally administered twice daily after meals for 14 consecutive days. Each capsule of S-1 contained 20 or 25 mg of tegafur. Individual doses were rounded to the nearest pill size less than the calculated dose, given the available formulation. Cisplatin was administered on days 1 and 8 via intravenous infusion for over 60 min. All the patients received prophylactic antiemetic therapy consisting of a 5-hydroxytryptamine 3 receptor antagonist, a neurokinin 1 receptor antagonist, and a steroid. The dose of each drug in this study was planned as follows: level 0, S-1–30 mg/m^2^ twice daily and cisplatin 40 mg/m^2^; and level 1, S-1–40 mg/m^2^ twice daily and cisplatin 40 mg/m^2^. Prophylactic administration of granulocyte colony-stimulating factors (G-CSF) was not permitted. G-CSF administration was permitted for patients with grade 4 and/or grade 3 febrile neutropenia.

#### 2.2.2 CIRT

**Figure 2:**
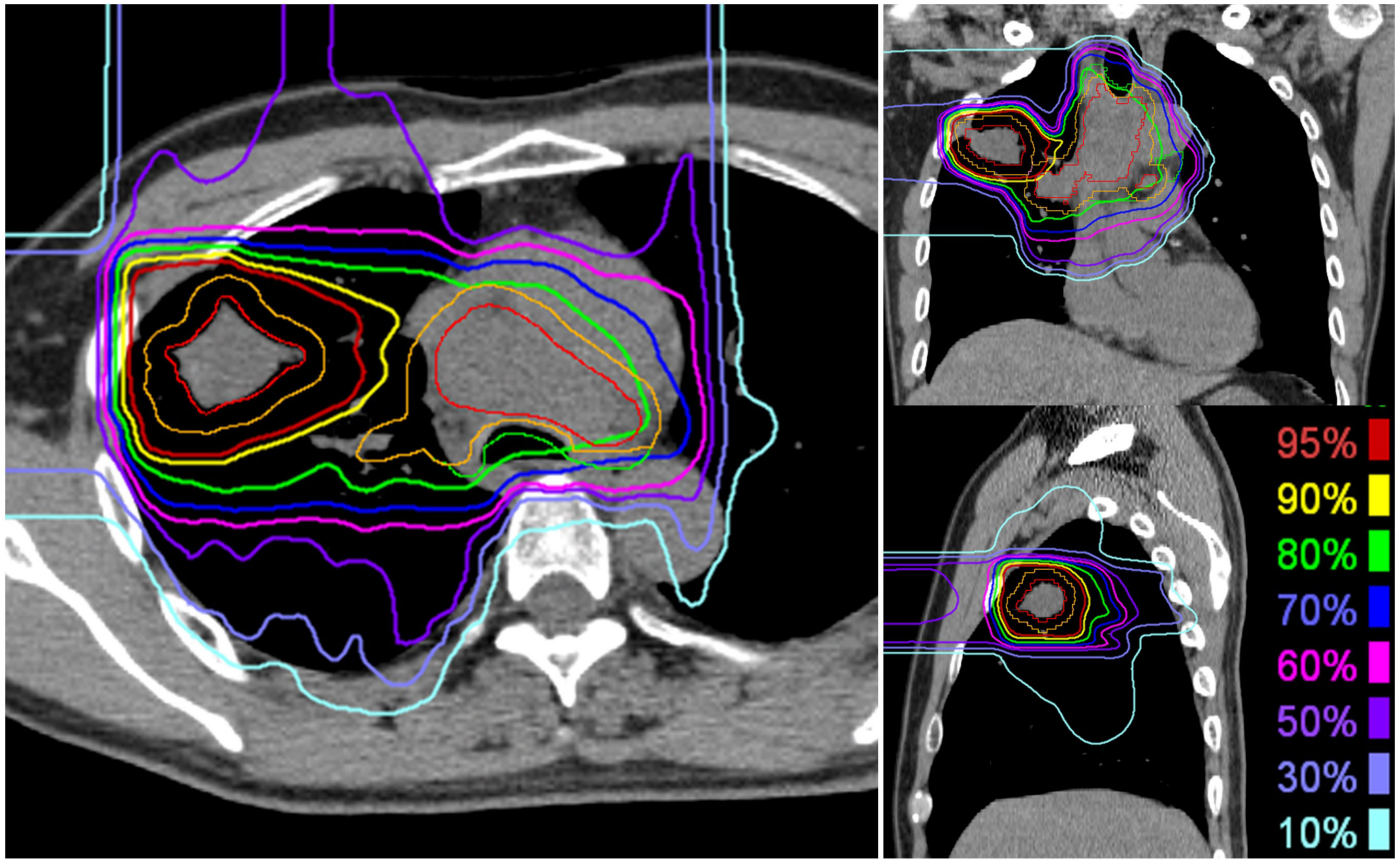
Representative dose distribution of carbon ion radiotherapy for locally advanced lung cancer. The primary tumor received a total dose of 64 Gy (RBE) delivered in 16 fractions, while lymph node metastases were irradiated with a total dose of 52 Gy (RBE). The gross tumor volume is delineated in red, and the clinical target volume for 52 Gy (RBE) is represented in orange.

The patients were immobilized in the supine or prone position using a thermoplastic shell (Shellfitter; Sanyo Polymer Industrial, Nara, Japan) and a pillow made of water-sclerogenic polymers (Moldcare; ALCARE, Tokyo, Japan). Computed tomography (CT) images were obtained using 2-mm-thick slices. A four-dimensional CT scan was obtained to quantify respiratory motion. Gross tumor volume (GTV) included the primary gross tumor and lymph node metastasis. The clinical target volume (CTV) was defined as the GTV plus a 5 mm margin in all directions, considering the anatomical boundaries. Planning target volume 64 (PTV64), which was irradiated at 64 Gy (RBE), was defined as the primary CTV with internal and setup margins. PTV52, which was irradiated at 52 Gy (RBE), was defined as primary CTV and lymph node metastases with internal and setup margins. When the PTV was close to organs at risk (OARs), such as the esophagus or spinal cord, the dose constraint of the OAR was prioritized over the target dose. The clinical dose distribution was calculated from the physical dose and RBE based on experimental results [5]. The unit of clinical dose was described as “Gy (RBE).” The passive scattering carbon-ion dose distribution was calculated using XiO-N (ELEKTA; Mitsubishi Electric, Tokyo, Japan). A total dose of 64 Gy (RBE) in 16 fractions was administered to the isocenter of the PTV for 4 weeks (4 fractions per week). In principle, the maximum dose to the OARs is defined as follows: spinal cord, 30 Gy (RBE); the esophagus, 60 Gy (RBE); trachea and main bronchus, 60 Gy (RBE); stomach or bowel, 40 Gy (RBE); and brachial plexus, 60 Gy (RBE). Daily orthogonal two-dimensional kV image pairs and weekly CT scans were used for image-guided radiotherapy. The dose distribution for a representative case with axial images is shown in **Figure 2**.

#### 2.2.3 Dose modification

The primary endpoint of the present study was to evaluate the recommended dose of SP and concurrent CIRT in patients with stage III LA-NSCLC. Doses were reduced from level 1 to level 0 according to the frequency of dose-limiting toxicity (DLT) evaluated between treatment initiation and 3 months after the last irradiation. Initially, six patients were treated with a dose of level 1. If fewer than two DLTs were observed in six patients, level 1 was determined to be the recommended dose (RD). If two DLTs were observed, discontinuation of patient entry at level 1 and recruitment of six patients at level 0 were planned. If fewer than two DLTs were observed in six patients, level 0 was determined as RD. If two DLTs were observed, study termination was planned. DLT was defined as: (1) ≥grade 3 nonhematologic toxicities except nausea/vomiting; (2) grade 4 thrombocytopenia; (3) grade 4 neutropenia; (4) Grade 3 or 4 febrile neutropenia; and (5) any unresolved toxicity requiring a delay in subsequent irradiation exceeding 14 days. Toxicities were assessed according to the Common Terminology Criteria for Adverse Events (CTCAE), version 5.0. Additional treatments, including consolidative durvalumab therapy, were prohibited until recurrence was diagnosed.

### 2.3 Assessment

Prior to treatment, the patients were evaluated using complete blood cell count, differential count, biochemical examinations, chest radiography, chest and abdominal contrast-enhanced CT, whole-brain magnetic resonance imaging or CT, and 18F-fluorodeoxyglucose positron emission tomography. Complete blood cell counts, differential counts, biochemical and physical examinations, chest radiography, and toxicity tests were performed weekly. Toxicity was graded according to CTCAE version 5.0. The tumor response was evaluated according to the Response Evaluation Criteria in Solid Tumor (RECIST) ver. 1.1 (16). Responses were defined as follows: complete response, disappearance of all target lesions; partial response, ≥30% reduction in size; stable disease, <30% decrease and <20% increase in size; and progressive disease, >20% increase in size (or the appearance of new lesions). The overall response was defined as the best response recorded from treatment initiation until disease progression or recurrence, confirmed by repeated assessments performed no less than 4 weeks after the criteria for response were first met. Survival was recorded from the first day of treatment with CIRT to the date of death or the last follow-up, and survival curves were prepared using the Kaplan–Meier method. OS was defined as the time from the date of the first administration of anticancer agents to death from any cause. PFS was defined as the time between the date of the first CIRT administration and the date of disease progression or death.

### 2.4 Statistical analysis

The Kaplan–Meier method was used to determine the actuarial survival rate. Statistical analyses were performed using GraphPad Prism 6 software (GraphPad Software, San Diego, CA, USA).

## 3 Results

### 3.1 Patients’ characteristics

Six patients, including four men and two women with a median age of 64 years (range, 49–69 years), were enrolled in this study at a dose of level 1 between September 2019 and September 2022. Patient characteristics are summarized in **Table 1**. ECOG PS was 0 in 2 patients and 1 in 4 patients. All the patients had a history of smoking. The histological types were adenocarcinoma in four patients and squamous cell carcinoma in two patients. The clinical stages were IIIA in 3 patients, IIIB in 1 patient, and IIIC in 2 patients. Comorbidities included chronic obstructive pulmonary disease in two patients and old myocardial infarction and chronic heart failure in one patient. Because RD was determined as level 1 as described below, no patients were enrolled at a dose of level 0.

**Table 1:** Patients’ characteristics and clinical outcomes.

Patient no.	Age	Gender	PS^#1^	TNM classification	Histological type	DLT^#2^	Toxicities (≥grade 2)	In-field recurrence	First recurrence site	PFS^#3^ (months)	OS^#4^ (months)	Survival
1	61	Female	1	T3N2M0	Adeno.^#5^	None	Esophagitis (G2)	None	Brain	8.5	43.7	Alive
2	61	Male	1	T2aN2M0	Sq.^#6^	Yes	Esophagitis (G2), lung infection (G2) elevated ALT/ALT(G3)	Yes (hilar/mediastinal lymph nodes)	Hilar/mediastinal lymph nodes, muscle, ventricle	5.0	21.7	Dead
3	49	Male	1	T4N3M0	Adeno.	None	Esophagitis (G2), dermatitis (G2)	None	Apex, supraclavicular node	9.5	32.0	Alive
4	69	Male	1	T4N3M0	Adeno.	None	Esophagitis (G2)	None	Brain	11.0	22.5	Alive
5	68	Male	0	T1cN2M0	Sq.	None	Esophagitis (G2), platelet count decreased (G2), white blood cell decreased (G2)	None	Mediastinal/supraclavicular lymph nodes	7.3	20.8	Alive
6	67	Female	0	T1cN2M0	Adeno.	None	Nausea (G2), anorexia (G2)	None	None	13.3	13.3	Alive

#1 PS, performance status. #2 DLT, dose limiting toxicities. #3 PFS, progression-free survival. #4 OS, overall survival. #5 Sq, squamous cell carcinoma. #6 adeno, adenocarcinoma.

### 3.2 Treatment delivery and toxicity

We evaluated toxicity in all treated patients. All adverse events are listed in **Table 2**. Six patients were enrolled in Level 1. Complete doses of chemotherapy and CIRT were administered to all patients. Patient #2 experienced grade 3 elevated ALT and AST levels 21 days after completion of the protocol. At the onset of elevated ALT and AST levels, loxoprofen and vonoprazan were administered to treat pain due to esophagitis. After the withdrawal of these drugs, ALT and AST levels decreased immediately. Although DLT was observed in this case, five patients did not develop DLTs. Five patients developed grade 2 esophagitis. In three of the five patients, symptoms such as pain and dysphagia due to esophagitis recurred several months after resolution of the acute esophagitis that occurred during irradiation. Patient #1 developed an esophageal ulcer 105 days after the completion of irradiation. Since the ulcer site was within the irradiation field, this event was considered treatment-related toxicity. The swallowing pain and other symptoms improved after a few months. None of the patients experienced ≥grade 3 pneumonitis or hematological toxicities. No treatment-related death occurred. Thus, level 1 was determined as the RD for the phase II study.

**Table 2:** Adverse events.

Adverse events	Grade				
	0	1	2	3	4
Leukopenia	1	4	1	0	0
Neutropenia	3	3	0	0	0
Anemia	1	4	1	0	0
Platelet count decreased	2	3	1	0	0
Elevated AST/ALT	4	0	1	1	0
Elevated creatinine	6	0	0	0	0
Nausea/vomiting	2	3	1	0	0
Anorexia	3	2	1	0	0
Constipation	5	1	0	0	0
Diarrhea	5	1	0	0	0
Esophagitis	1	0	5	0	0
Fatigue	6	0	0	0	0
Fever	3	3	0	0	0
Febrile neutropenia	6	0	0	0	0
Infection	5	0	1	0	0
Alopecia	6	0	0	0	0
GI-hemorrhage	6	0	0	0	0
Pneumonia	1	4	1	0	0
Dermatitis radiation	1	4	1	0	0
Hiccups	5	1	0	0	0
Myelitis	6	0	0	0	0

AST, aspartate transaminase; ALT, alanine transaminase; GI, Gastrointestinal.

### 3.3 Response and survival

The clinical courses of the six patients are shown in **Table 1**. All patients exhibited at least a partial response. One patient developed in-field recurrence, and two patients developed metastases to the lymph nodes around the PTV. Two patients (Patients #3 and #5) underwent CIRT for recurrence. Patient #3 was free of recurrence 20 months after the second CIRT for the apex and supraclavicular nodes. Patient #5 experienced multiple lung metastases 4 months after the completion of the second CIRT for the mediastinal/supraclavicular lymph nodes. Distant metastases were observed in three patients. The median PFS and OS were 8.5 months and not reached, respectively, and the median follow-up period was 22.1 months. The data cutoff date for this study was December 2023.

### 3.4 Dosimetric analysis

**Table 3** lists the dosimetric parameters for all patients. A mean PTV dose of 64 Gy was almost achieved at the prescribed dose of 64 Gy (RBE). Patient #3 had a tumor that invaded the spinal canal and was adjacent to the spinal cord; hence, the doses of the PTV64 and GTV primary tumors were low compared to those of the others. However, the patient did not experience local recurrence near the spinal cord. The mean V5 and V20 for lung-GTV were 20.4% and 15.6%, respectively. All patients achieved D0.7cc for the spinal cord under 30 Gy (RBE). Patients #1, #3, and #5, who had recurrent esophagitis, had an esophageal D1cc greater than 50 Gy (RBE).

**Table 3:** Dosimetric analysis of carbon ion radiotherapy.

Case	PTV 64			PTV52		
	Volume (ml)	D95 (Gy [RBE])	Mean (Gy [RBE])	Volume (ml)	D95 (Gy [RBE])	Mean (Gy [RBE])
1	35.3	63.4	64.2	291.8	51.2	53.7
2	57.9	61.7	63.9	349.1	50.1	54.4
3	300.4	52.7	62.5	343.1	52.4	61.8
4	290.7	60.1	63.7	418.1	52.1	60.7
5	79.5	63.3	64.3	173.8	50.8	57.8
6	43.6	63.0	64.5	91.2	52.4	59.6
Mean (SD)	134.6 (125.6)	60.7 (4.1)	63.8 (0.7)	277.9 (122.4)	51.5 (1.0)	58.0 (3.3)
Case	GTV primary			GTV Lymph node		
	Volume (ml)	D95 (Gy [RBE])	Min (Gy [RBE])	Volume (ml)	D95 (Gy [RBE])	Min (Gy [RBE])
1	3.8	64.2	64.0	70.7	51.9	50.8
2	11.8	63.2	62.6	95.2	51.4	45.6
3	139.0	53.2	33.6	10.8	53.1	52.5
4	126.5	64.0	57.3	13.8	52.3	51.8
5	18.0	63.9	63.6	10.6	51.0	50.3
6	10.4	63.1	58.4	8.6	52.4	52.1
Mean (SD)	51.6 (63.2)	61.9 (4.3)	56.6 (11.6)	34.9 (38.0)	52 (0.7)	50.5 (2.5)
Case	Trachea, main bronchus	Lung-GTV				
	Max (Gy [RBE])	Mean (Gy [RBE])	V5 (%)	V10 (%)	V20 (%)	V30 (%)
1	53.8	10.6	29.0	25.7	20.8	17.7
2	54.2	10.0	31.6	28.4	20.6	15.0
3	57.6	2.8	7.2	6.6	5.6	4.5
4	55.9	8.3	17.5	16.6	15.4	14.2
5	52.9	8.3	19.2	17.9	16.2	13.8
6	55.9	7.7	17.8	16.7	15.1	12.9
Mean (SD)	55.1 (1.7)	7.9 (2.7)	20.4 (8.8)	18.7 (7.7)	15.6 (5.5)	13.0 (4.5)
Case	Spinal cord		Esophagus				
	Max (Gy [RBE])	D0.7cc (Gy [RBE])	Max (Gy [RBE])	D1cc (Gy [RBE])		D2cc (Gy [RBE])	
1	24.3	16.9	53.3	50.6		48.3	
2	14.3	4.8	49.5	44.6		43.5	
3	39.4	27.6	56.7	53.0		52.8	
4	33.7	29.6	52.1	47.6		45.7	
5	24.2	22.6	52.5	52.0		51.8	
6	23.2	21.8	31.7	2.1		0.3	
Mean (SD)	26.5 (8.8)	20.5 (8.9)	49.3 (8.9)	41.7 (19.6)		40.4(19.9)	

PTV, planning target volume; GTV, gross target volume; D n, minimum dose to maximally irradiated n; Vn, Total volume irradiated for > n Gy; RBE, relative biological effectiveness; min, minimum; max, maximum.

## 4 Discussion

This is the first phase I study to evaluate the tolerability of concurrent chemotherapy with SP and CIRT in patients with inoperable stage III NSCLC. This study demonstrated that one patient experienced DLT, and RD was defined as level 1. We observed that the treatment was tolerable. There were no ≥grade 3 toxicities other than elevated AST and ALT. Other notable findings are the high frequency of grade 2 esophagitis and the delayed onset of recurrent esophagitis.

Five of the six patients experienced recurrence outside the irradiated fields, and the effect of the combination of CIRT with SP in suppressing potential distant metastasis appeared to be insufficient. A possible reason for this may be that the short duration of the CIRT treatment resulted in the administration of one course of SP when used only within the concurrent use period, in contrast to two to four courses of SP for conventional radiotherapy without consolidation therapy with durvalumab (11–14). Future studies on the use of durvalumab maintenance therapy to prevent distant metastases are warranted.

Local control remains a challenge in cCRT and radiography for stage III NSCLC. The RTOG 0617 trial, which compared standard- and high-dose chemoradiotherapy, reported that about half of the patients experienced locoregional recurrence (17). Another phase III trial of cCRT reported that one-third of patients had local recurrence (18, 19). The PACIFIC trial also reported that the lungs and lymph nodes were the most common sites of recurrence (3). For CIRT without chemotherapy for locally advanced NSCLC, a study reported a 2-year local control rate of 73.9–100% (6–8). In our study, only one of six patients experienced recurrence within the irradiation field. The effectiveness of the addition of SP to CIRT for local control was not clear because of the high local control rate of CIRT alone. In the future, it is expected that a combination of immune checkpoint inhibitors will help to take advantage of the local control of CIRT if distant metastasis is reduced.

Although the standard treatment for stage III lung cancer is 1 year of consolidation therapy with durvalumab after cCRT, not all patients can complete the full course. In the PACIFIC study, only 49% of the patients who received durvalumab completed 1 year of treatment. Of the durvalumab discontinuations, 30% were due to adverse events (20). Additionally, 23–55% of patients with stage III NSCLC treated with cCRT did not meet the criteria of the PACIFIC trial, and some of these patients could not be initiated on durvalumab (21, 22). Approximately 20% of the reasons for not initiating durvalumab were due to radiation pneumonitis (23). The risk of ≥grade 3 pulmonary toxicities in stage III lung cancer with cCRT is 7–20.6% (17, 18). Taking these considerations into account, reducing radiation-induced pulmonary toxicity is important for continuing or initiating durvalumab. The radiation dose to lung tissues is associated with the incidence of radiation pneumonitis (24, 25). Theoretically, CIRT can reduce lung doses compared to X-rays (4); this might reduce pulmonary toxicity. In fact, two prospective clinical trials have reported that the incidence of ≥grade 3 radiation pneumonitis was 0–1.6%, which is lower than that in patients treated with conventional CRT (6, 7). This could increase the accessibility of durvalumab consolidation therapy and potentially improve clinical outcomes. In our study, one case of pneumonia occurred but improved with antibiotics over approximately 2 weeks without the need for steroids, suggesting no radiation-induced pneumonitis.

Our study had some limitations. First, our study could not verify the efficacy of concurrent chemotherapy with SP and CIRT owing to the nature of a phase I study with a small sample size. Second, this study did not permit consolidative durvalumab therapy after protocol treatment. Given the tolerability of concurrent chemotherapy and CIRT in the present study, prospective evaluation of the efficacy and tolerability of this treatment, followed by durvalumab consolidation, is warranted in the future.

In conclusion, our study demonstrated that concurrent chemotherapy with SP and CIRT is tolerable. The RD for the phase II study was S-1–40 mg/m^2^ twice daily for 14 consecutive days and cisplatin 40 mg/m^2^ on days 1 and day 8. Prospective studies are warranted to examine the efficacy of this treatment.

## Data Availability

The raw data supporting the conclusions of this article will be made available by the authors, without undue reservation.
